# Usp8 promotes tumor cell migration through activating the JNK pathway

**DOI:** 10.1038/s41419-022-04749-1

**Published:** 2022-03-31

**Authors:** Yunhe Zhao, Dezhen Peng, Yanyun Liu, Qian Zhang, Bin Liu, Yanran Deng, Wenhao Ding, Zizhang Zhou, Qingxin Liu

**Affiliations:** 1 State Key Laboratory of Crop Biology, College of Life Sciences, Shandong Agricultural University, 271018 Tai’an, China; 2 Jiangsu Key Laboratory of Drug Screening, China Pharmaceutical University, 210009 Nanjing, China

**Keywords:** Metastasis, Cell migration

## Abstract

Tumor metastasis is the most cause of high mortality for cancer patients. Identification of novel factors that modulate tumor cell migration is of great significance for therapeutic strategies. Here, we find that the ubiquitin-specific protease 8 (Usp8) promotes tumor cell migration through activating the c-Jun N-terminal kinase (JNK) pathway. Genetic epistasis analyses uncover Usp8 acts upstream of Tak1 to control the JNK pathway. Consistently, biochemical results reveal that Usp8 binds Tak1 to remove ubiquitin modification from Tak1, leading to its stabilization. In addition, human USP8 also triggers tumor cell migration and activates the JNK pathway. Finally, we show that knockdown of USP8 in human breast cancer cells suppresses cell migration. Taken together, our findings demonstrate that a conserved Usp8-Tak1-JNK axis promotes tumor cell migration, and providing USP8 as a potential therapeutic target for cancer treatment.

## Introduction

Cancer has become the leading cause of human death worldwide [1]. Tumor metastasis, rather than the primary tumor overgrowth, is the main cause of tumor-induced mortality [2]. Tumor metastasis is a complex process controlled by various genes, and its underlying mechanism still remains unclear [2]. Therefore, exploring novel regulator of tumor cell migration is of great significance for therapeutic strategies. During past decades, wing disc has emerged as an ideal model for studying tumor cell migration in *Drosophila* [3]. To establish a tumor migration model, the cell polarity gene *scribbled* (*scrib*) is silenced along the anterior/posterior compartment boundary in wing discs using *ptc-gal4* driver. Knockdown of *scrib* confers wing disc cells invasive characteristics [4].

As a member of the mitogen-activated protein kinase (MAPK) family, the evolutionarily conserved JNK pathway is involved in various cellular processes, such as apoptosis, migration, proliferation, and differentiation [5, 6]. The JNK pathway is a kinase cascade which is activated by diverse extrinsic and intrinsic stimuli. In *Drosophila*, the ligand Eiger (Egr) binds its receptors Wengen (Wgn) or Grindelwald (Grnd) to trigger the downstream JNKKKs, including transforming growth factor-*β*-associated kinase 1 (Tak1), Slipper (Slpr), Wallenda (Wnd) and apoptotic signal-regulating kinase 1 (Ask1). In turn, JNKKKs activate the downstream JNKKs, including Hemipterous (Hep) and Mkk4, which sequentially phosphorylate and activate the *Drosophila* sole JNK, Basket (Bsk) [7]. Activated JNK phosphorylates the putative transcriptional factor Jun to promote its nuclear accumulation [6]. One of well-documented Jun target genes is *matrix metalloproteinase 1* (*mmp1*), which encodes a proteinase that hydrolyzes proteins in the extracellular matrix to trigger cell migration [8]. Although it is clear that phosphorylation modification is important for maintaining the JNK pathway homeostasis, roles of other post-translational modifications (PTMs), such as ubiquitination, in this pathway still remain poorly understood.

The process of ubiquitination requires three classes of enzymes: E1 ubiquitin-activating enzymes, E2 ubiquitin-conjugating enzymes, and E3 ubiquitin ligases [9]. Through the coordinated action of these enzymes, the first 76-amino-acid polypeptide ubiquitin (Ub) is covalently attached to the lysine residue (K) of the substrate [9]. Next, the following Ub is attached to one of the seven lysine residues of the previous Ub to form polyubiquitin chain. Distinct linkage of polyubiquitin chain will lead to diverse fates of substrates [10]. Recently, increasing studies have revealed several components of JNK pathway undergo ubiquitination modification, including Traf2 [11], Tak1 [12], Ask1 [13], and Bsk [14]. As a matter of fact, ubiquitination is also a reversible process due to a class of enzymes named deubiquitinases, which could remove ubiquitin chains from substrates [15]. Usp8, also known as Ubpy, is an evolutionarily conserved ubiquitin-specific protease [16]. Usp8 is able to remove K6-, K48-, and K63-linked polyubiquitin chains from substrates, thus plays roles in multiple cellular processes [17]. Several substrates of Usp8 have been identified, including Smoothened (Smo) [18, 19], Hrs [20], *α*-Synuclein [21], and CHMP1B [22]. Furthermore, previous studies have demonstrated that elevated USP8 expression is associated with a poor prognosis in various types of cancers, suggesting that USP8 contributes to tumorigenesis [23, 24]. Albeit the JNK pathway is a key regulator of tumor migration, whether and how Usp8 modulates this pathway is still unclear.

Here, we found that overexpression of Usp8 promoted *scrib*-RNAi-caused tumor cell migration, while knockdown of *usp8* exerted an opposite effect. Next, we uncovered that Usp8 was capable of activating the JNK pathway, and inhibition of the JNK pathway could suppress Usp8-enhanced tumor cell migration. Genetic analyses showed that Usp8 localized upstream of Tak1 to activate the JNK pathway. Further biochemical results revealed that Usp8 interacted with Tak1 to deubiquitinate it, culminating in Tak1 stabilization. Human USP8 also promoted *scrib*-RNAi-caused tumor cell migration and activated the JNK pathway in *Drosophila* wing discs, suggesting a conserved role of USP8 in regulating JNK signaling in mammalian system. Finally, we demonstrated that knockdown of *USP8* in human breast cancer cells suppressed cell migration and downregulated the JNK pathway activity. Taken together, our study uncovers a conserved Usp8-Tak1-JNK axis to promote tumor cell migration, and provides USP8 as a potential therapeutic target for JNK-related cancers.

## Results

### Usp8 is able to trigger cell migration

**Fig. 1:**
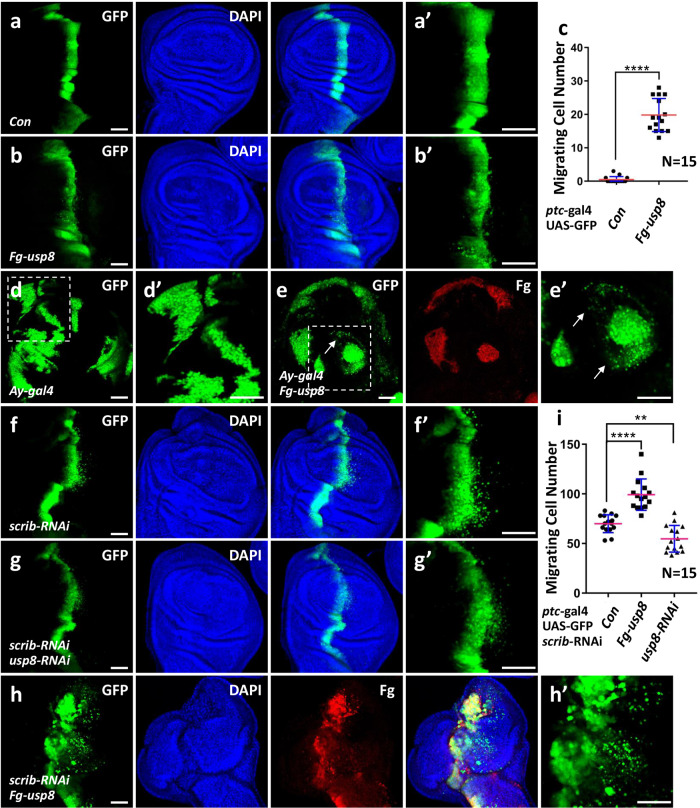
Ectopic expression of Usp8 induces cell migration. **a** The third-instar larvae wing disc of *ptc*-gal4 was used as a control, **a'** was an enlarged figure of **a**. **b** Overexpression of *usp8* driven by *ptc*-gal4 induced cell migration from A/P boundary to posterior. GFP (green) marks the expression regions of *ptc*-gal4 in the wing disc. **c** Quantification analyses of migrating cell numbers in **a,** **b** (*N* = 15). **d** A control wing disc expressing GFP using FLP-out technique was stained with GFP (green). **e** Ectopic expression of *usp8* in the wing disc through FLP-out technique triggered cell migration (arrows). **f** Knockdown of *scrib* in the wing disc induced cell migration. **g** Knockdown of *usp8* suppressed *scrib*-RNAi-induced cell migration. **h** Overexpression of *usp8* promoted *scrib*-RNAi-induced cell migration. **i** Quantification analyses of migrating cell numbers in **f**–**h** (*N* = 15). All wing imaginal discs shown in this study were oriented with anterior on the left and ventral on the top. The *t*-test was used for statistical analyses, **p < 0.01; ****p < 0.0001. Scale bars: 50 μm for all disc images.

To explore the roles of deubiquitinases in regulating tumor cell migration, we generated transgenic flies expressing *Drosophila* deubiquitinases. Then, the transgene was ectopically expressed driven by *patched*-gal4 (*ptc*-gal4), which expresses along the anterior/posterior (A/P) compartment boundary in the wing disc. After screening, we found that overexpression of *usp8* induced mild cell migration (Fig. 1b, c) compared to the control disc (Fig. 1a, c). To show the role of Usp8 in cell migration is not confined to the A/P boundary cells, we randomly expressed *usp8* in the wing disc using the FLP-out technique and marked the *usp8*-expressing cells with green fluorescence (GFP). Compared with the control (Fig.1d), overexpression of *usp8* was able to activate cell migration (Fig. 1e).

The previous study has demonstrated that knockdown of the cell polarity gene *scrib* in the wing disc induces an invasive migration phenotype, which has been widely used as model tumor cell metastasis [4, 25]. To investigate whether Usp8 is involved in regulating tumor cell migration, we overexpressed or silenced *usp8* under *scrib* RNAi background. Consistently, *scrib* RNAi indeed triggered invasive cell migration (Fig. 1f, i), which was attenuated by *usp8* knockdown (Fig. 1g, i), whereas aggravated by *usp8* overexpression (Fig. 1h, i). Taken together, these results show that Usp8 is able to trigger cell migration.

### Usp8 activates the JNK pathway to promote tumor cell migration

**Fig. 2:**
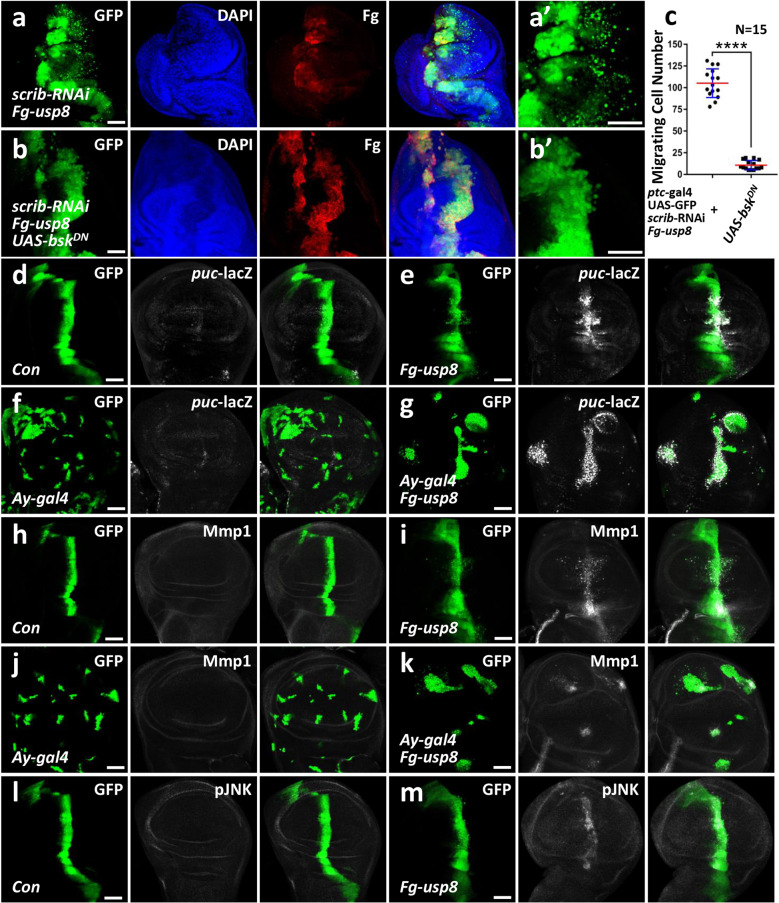
Usp8 triggers cell migration through activating the JNK pathway. **a** Ectopic expression of *usp8* under *scrib* RNAi background induced robust cell migration in the wing disc. **a’** was an enlarged figure of **a**. **b** Overexpression of *bsk*^*DN*^ blocked *usp8*-induced cell migration under *scrib* RNAi background. **c** Quantification analyses of migrating cell numbers in **a, b** (*N* = 15). **d** A control third-instar larvae wing disc expressing GFP under *ptc*-gal4 driver was stained to show GFP (green) and *puc*-lacZ (white). **e** Overexpression of *usp8* increased *puc*-lacZ level. **f** A control wing disc expressing GFP via FLP-out technique was stained with GFP (green) and *puc*-lacZ (white). **g** Ectopic expression of *usp8* in the wing disc by FLP-out technique elevated *puc*-lacZ level. **h** A control wing disc expressing GFP under *ptc*-gal4 driver was stained to show GFP (green) and Mmp1 (white). **i** Overexpression of *usp8* increased Mmp1 protein. **j** A control wing disc expressing GFP via FLP-out technique was stained with GFP (green) and Mmp1 (white). **k** Ectopic expression of *usp8* in the wing disc by FLP-out technique elevated Mmp1 protein level. **l** A control wing disc expressing GFP was stained with GFP (green) and pJNK (white). **m** Overexpression of *usp8* in the wing disc by *ptc*-gal4 elevated pJNK level. The *t*-test was used for statistical analyses, ****p < 0.0001. Scale bars: 50 μm for all disc images.

Given that the JNK pathway has been recognized as an important mechanism controlling cell migration through its target Mmp1 in *Drosophila* [8], we first examined whether the JNK pathway is required for Usp8 to promote tumor cell migration. In the *Drosophila*, various upstream stimuli activate a kinase cascade to converge on the sole Jun kinase Basket (Bsk), leading to activation of the JNK pathway [6]. We used a dominant negative form of Bsk (Bsk^DN^), in which the lysine on 53 is substituted by an arginine to suppress the endogenous JNK signaling [26]. Compared with *usp8* alone (Fig. 2a, c), overexpression of *bsk*^*DN*^ effectively inhibited the enhanced tumor cell migration induced by *usp8* expression (Fig. 2b, c), indicating that the JNK pathway is required for Usp8-mediated tumor cell migration.

In our above screening, we found that ectopic expression of *usp8* using *ptc*-gal4 resulted in loss of the anterior cross-vein (ACV) phenotype in adult wings (Supplementary Fig. 1a–e), which is an indicator for JNK signaling activation [27]. To further examine whether Usp8 is able to activate the JNK pathway, we checked the expression of *puc*-lacZ, a widely used readout of this pathway [28]. Compared with the control disc (Fig. 2d), overexpression of *usp8* by *ptc*-gal4 driver robustly increased *puc*-lacZ expression (Fig. 2e). To further substantiate this result, we generated random clones transiently expressing *usp8* using FLP-out technique and found that Usp8 indeed elevated *puc*-lacZ expression in these clones (Fig. 2g) compared to control clones (Fig. 2f). In addition, we showed that overexpression of *usp8* using *ptc*-gal4 (Fig. 2h, i) or FLP-out technique (Fig. 2j, k) also activated the expression of Mmp1, another well-documented JNK pathway target. JNK phosphorylation is a critical step for this pathway activation, and JNK phosphorylation level (pJNK) serves as a signal for JNK signaling activation [29]. Compared with the control disc (Fig. 2l), ectopic expression of *usp8* by *ptc*-gal4 driver apparently upregulated pJNK (Fig. 2m). To test whether the regulation of Usp8 on the JNK pathway is also applicable to other tissues, we overexpressed usp8 in eye discs. Compared with the control (Supplementary Fig. 2a), ectopic expression of *usp8* in the eye disc by *mirr*-gal4 driver also activated *puc*-lacZ expression (Supplementary Fig. 2b). In addition, Usp8 was able to increase Mmp1 expression in the eye disc (Supplementary Fig. 2c, d). The FLP-out technique further confirmed that both *puc*-lacZ and Mmp1 were upregulated in *usp8*-expressing clones in eye discs (Supplementary Fig. 2e–h). Overall, these results support that Usp8 activates the JNK pathway to promote tumor cell migration.

### Usp8 acts downstream of Traf2, upstream of Tak1

**Fig. 3:**
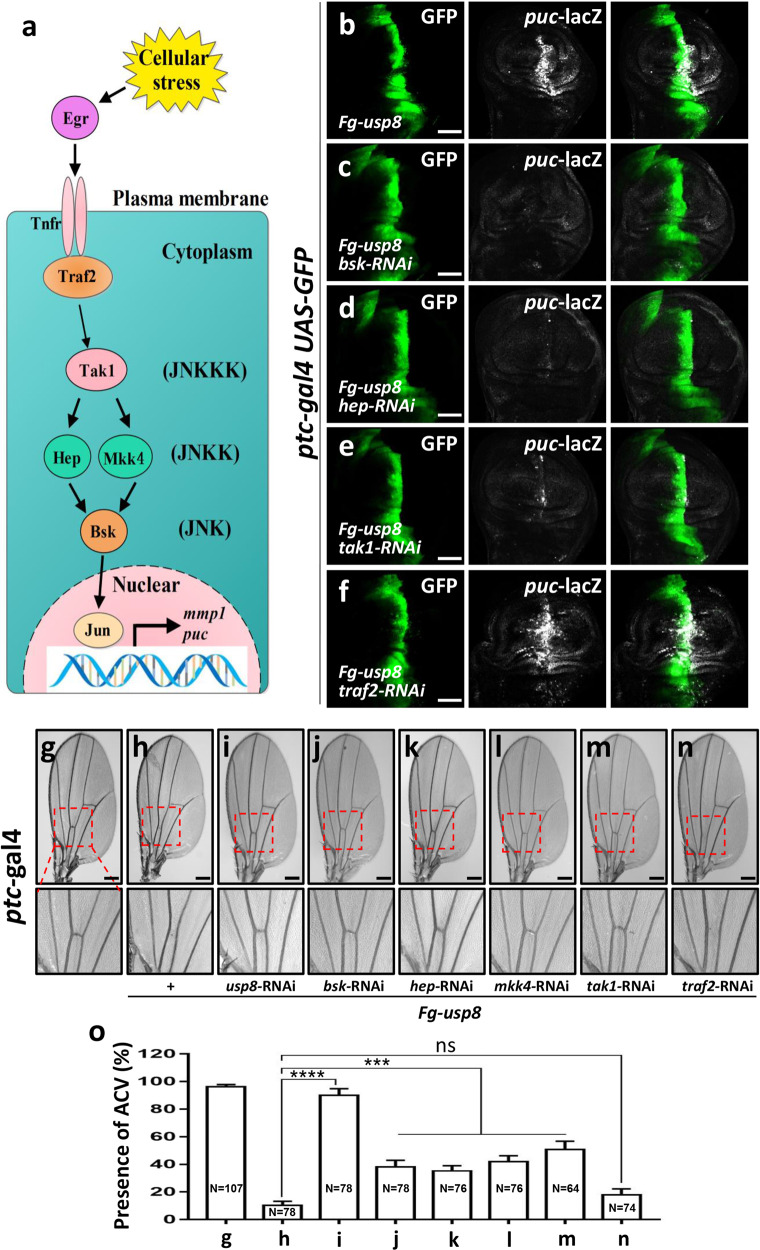
Usp8 acts downstream of Traf2, and upstream of Tak1. **a** Simplified schematic illustrating the cascade of JNK pathway. Egr, Eiger; Tnfr, Tumor necrosis factor receptors; Traf2, TNF-receptor-associated factor 2; Tak1, TGF-*β*-associated kinase 1; Hep, Hemipterous; Mkk4, MAP kinase kinase 4; Bsk, Basket; *puc*, puckered; *mmp1*, matrix metalloproteinase 1. **b** A wing disc with *usp8* ectopic expression by *ptc*-gal4 was stained to show GFP (green) and *puc*-lacZ (white). **c** Knockdown of *bsk* inhibited Usp8-induced *puc*-lacZ upregulation. **d** Knockdown of *hep* attenuated *puc*-lacZ expression caused by *usp8* ectopic expression. **e** Knockdown of *tak1* inhibited *puc*-lacZ expression caused by *usp8* ectopic expression. **f** Knockdown of *traf2* failed to decrease *puc*-lacZ expression caused by *usp8* ectopic expression. **g** The adult wing of *ptc*-gal4 was used as a control. **h** Overexpression of *usp8* resulted in the loss of ACV phenotype. **i**-**n** The loss of ACV caused by *usp8* overexpression is rescued by depletion of *usp8* (**i**), or *bsk* (**j**), or *hep* (**k**), or *mkk4* (**l**), or *tak1* (**m**), but not *traf2* (**n**). **o** Statistical analyses of the presence of ACV in **g**–**n** were presented as mean ± SD. Numbers of wings for statistical analyses were shown in columns. Above all, the *t*-test was used for statistical analyses, ns (not significant), *p* > 0.05; ***p < 0.001; ****p < 0.0001. Scale bars: 50 μm for all disc images, 200 μm for all adult wing images.

Activation of the JNK pathway involves a kinase cascade that ultimately converges on the transcriptional factor Jun to control the expression of target genes [6] (Fig. 3a). Given that Usp8 is able to activate the JNK pathway, we next sought to explore the underlying mechanism. Genetic epistasis experiments revealed that the upregulated *puc*-lacZ induced by *usp8* overexpression (Fig. 3b) could be restored by *bsk* RNAi (Fig. 3c), *hep* RNAi (Fig. 3d), or *tak1* RNAi (Fig. 3e), while failed to be rescued by *traf2* RNAi (Fig. 3f), indicating that Usp8 localizes downstream of Traf2, but upstream of Tak1, to modulate the JNK pathway. In addition, we also employed the loss of ACV phenotype in adult wings to confirm the epistatic result. Compared with the control wing (Fig. 3g, o), ectopic expression of *usp8* by *ptc*-gal4 driver resulted in loss of the ACV phenotype (Fig. 3h, o), which was partially rescued by knockdown of *bsk* (Fig. 3j, o), *hep* (Fig. 3k, o), *mkk4* (Fig. 3l, o), or *tak1* (Fig. 3m, o), but not *traf2* RNAi (Fig. 3n, o). Hence, we provide evidence to support that Usp8 acts downstream of Traf2, but upstream of Tak1, to activate the JNK pathway.

### Usp8 interacts with Tak1

**Fig. 4:**
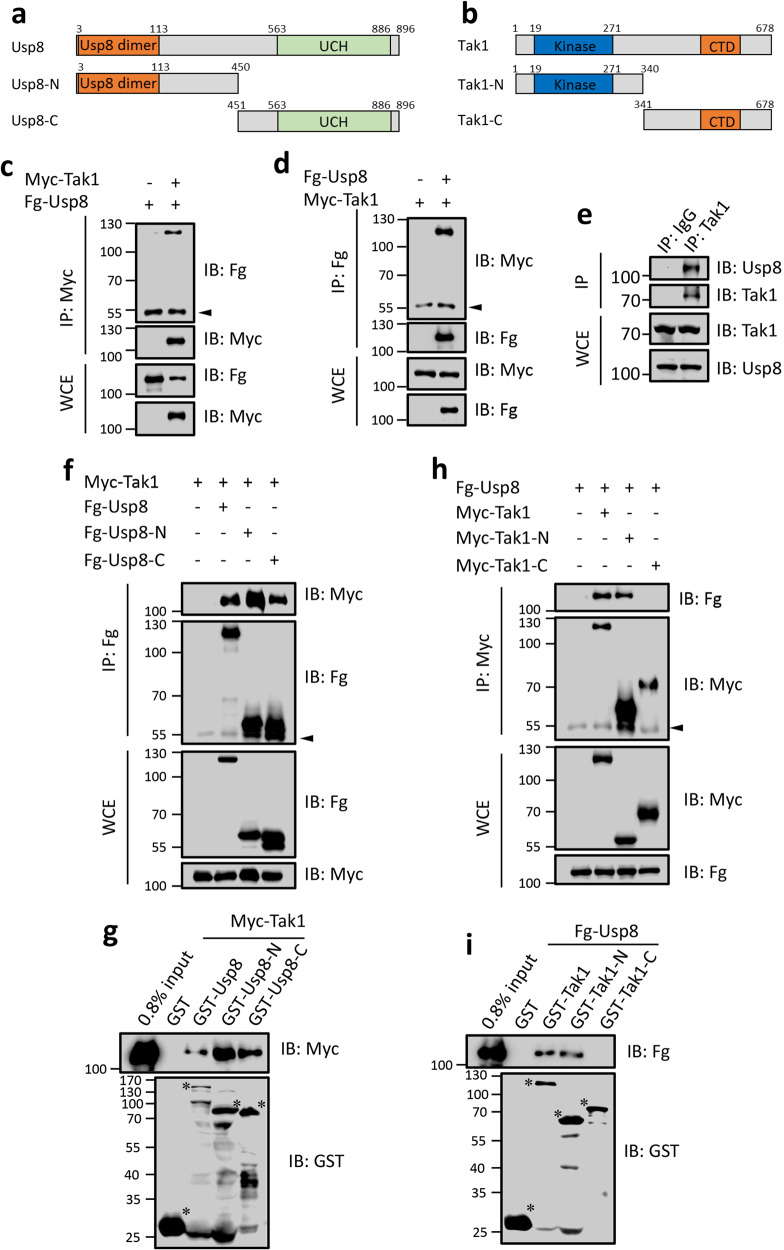
Usp8 interacts with Tak1. **a**, **b** The schematic diagrams show the domains in Usp8 and Tak1, and their truncated constructs used in the following co-IP and GST pull-down assays. Orange and green bars represent the Usp8 dimerization domain (Usp8 dimer) and ubiquitin carboxyl-terminal hydrolase domain (UCH) of Usp8, respectively. Blue and orange bars indicate the kinase domains and C-terminal domain (CTD) of Tak1, respectively. **c** Myc-Tak1 could pull down Fg-Usp8 in 293 T cells. The arrowhead indicates the IgG. **d** Fg-Usp8 pulled down Myc-Tak1 in 293 T cells. **e** In the third instar larval stage, endogenous Tak1 interacted with endogenous Usp8. **f** Myc-Tak1 interacted with Fg-tagged N- and C-fragments of Usp8. **g** GST pull-down between Myc-Tak1 and GST or GST-tagged Usp8 truncated constructs. Asterisks mark expressed GST fusion proteins. **h** Tak1 interacted with Usp8 through its N-terminal region in 293 T cells. **i** GST pull-down between Fg-Usp8 and GST or GST-tagged Tak1 truncated constructs.

The deubiquitinase always exerts its biological function through binding and removing ubiquitin chains from the substrate. Therefore, we speculated that Usp8 possibly interacts with one component of the JNK pathway. Since the above epistasis results show that Usp8 localizes upstream of Tak1, we first examined the interaction between Usp8 and Tak1 through co-immunoprecipitation (co-IP) experiments. The co-IP result revealed that Myc-tagged Tak1 (Myc-Tak1) was able to pull down Fg-tagged Usp8 (Fg-Usp8) (Fig. 4c). Reciprocally, Fg-Usp8 also pulled down Myc-Tak1 (Fig. 4d), but not other major components of JNK pathway (Supplementary Fig. 3a–f). To confirm the interaction between endogenous Usp8 and Tak1, we generated mouse anti-Usp8 antibody and mouse anti-Tak1 antibody. The western blot results showed that these two antibodies worked well (Supplementary Fig. 4a, b). Consistently, using the third instar larval protein samples, endogenous Tak1 pulled down endogenous Usp8 (Fig. 4e). Taken together, these results suggest that there is an interaction between Usp8 and Tak1.

Usp8 protein contains a Usp8 dimerization domain (Usp8 dimer) domain in its N-terminus, a ubiquitin carboxyl-terminal hydrolase (UCH) domain in its C-terminus (Fig. 4a). To determine which domain in Usp8 is responsible for its interaction with Tak1, we generated two truncated constructs (Fig. 4a). The co-IP result showed that both N-terminus and C-terminus were able to bind Tak1 (Fig. 4f). The GST pull-down assay further validated this result (Fig. 4g). On the other hand, to map the Usp8-binding domain in Tak1, two truncated constructs were used to express the N-terminus (Tak1-N) and C-terminus (Tak1-C) of Tak1, respectively (Fig. 4b). Co-IP and GST pull-down assays revealed that Tak1-N could bind Usp8, while Tak1-C failed to do (Fig. 4h, i).

### Usp8 deubiquitinates and stabilizes Tak1

**Fig. 5:**
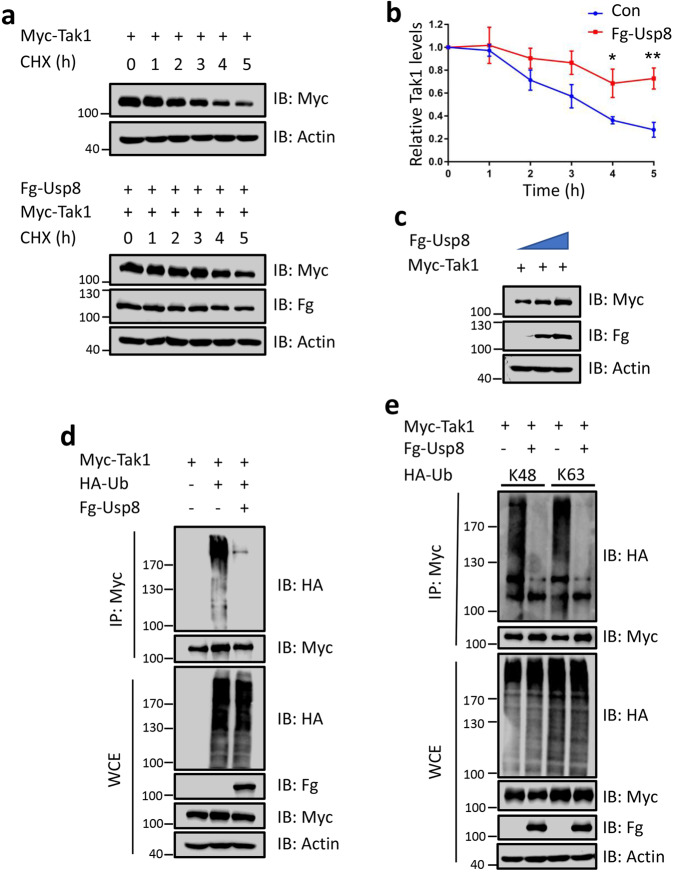
Usp8 deubiquitinates and stabilizes Tak1. **a** 293 T cells were transfected with Myc-Tak1 or Myc-Tak1 together with Fg-Usp8 and treated with cycloheximide (CHX) for indicated times. Then, immunoblots of these lysates were conducted. **b** Quantification analyses of relative Tak1 protein levels were shown (*N* = 3). Notably, Usp8 effectively inhibited Tak1 degradation. **c** Immunoblots of lysates of 293 T cells transfected with indicated constructs. Of note, Usp8 stabilizes Tak1 protein in a dose-dependent manner. **d** Immunoblots of immunoprecipitates and lysates from 293 T cells expressing indicated constructs and treated by MG132 (50 μM) for 4 h before cell harvesting. Notably, Usp8 attenuated Tak1 ubiquitination. **e** Usp8 hampered Lys48- and Lys63-linked polyubiquitination on Tak1. Above all, Actin acts as a loading control. The *t*-test was used for statistical analyses, *p < 0.05; **p < 0.01.

The interaction between Usp8 and Tak1 prompted us to examine whether Tak1 is a substrate of Usp8. First, we used the translation inhibitor cycloheximide (CHX) to block protein synthesis and determine the stability of Tak1 with or without Usp8 co-expression. Strikingly, Tak1 protein was unstable, with a half-life of about 3 h (Fig. 5a, b). However, the half-life of Tak1 was prolonged in the presence of Usp8 (Fig. 5a, b). Meanwhile, we found that Usp8 could stabilize Tak1 in a dose-dependent manner (Fig. 5c). Next, we examined whether Usp8 was able to decrease ubiquitination of Tak1. Cell-based ubiquitination experiments showed that Usp8 indeed diminished Tak1 ubiquitination (Fig. 5d), together suggesting that Usp8 binds and deubiquitinates Tak1. As a matter of fact, previous studies have demonstrated Tak1 protein undergoes distinct linkages of polyubiquitination. The K63-linked polyubiquitination on Tak1 is required for its autophosphorylation and subsequent activation [12], while K48-linked polyubiquitination promotes proteasome-mediated Tak1 degradation [30]. Since the previous report has shown that Usp8 is capable of removing both K48- and K63-linked polyubiquitin chains from substrates [17], we next wanted to test which linkage of polyubiquitination on Tak1 protein is sheared by Usp8. To this end, we generated two mutant Ub constructs, Ub-K48 and Ub-K63, which harbors only one K on 48 or 63, respectively. Thus, Ub-K48 only produces K48-linked polyubiquitin chains, whereas Ub-K63 forms K63-linked polyubiquitin chains. Our results demonstrated that Usp8 was able to decrease both K48- and K63-polyubiquitination on Tak1 (Fig. 5e). Collectively, these results suggest that Usp8 binds Tak1 to deubiquitinate Tak1, culminating in Tak1 stabilization.

### Human USP8 can replace *Drosophila* Usp8 to modulate the JNK pathway

**Fig. 6:**
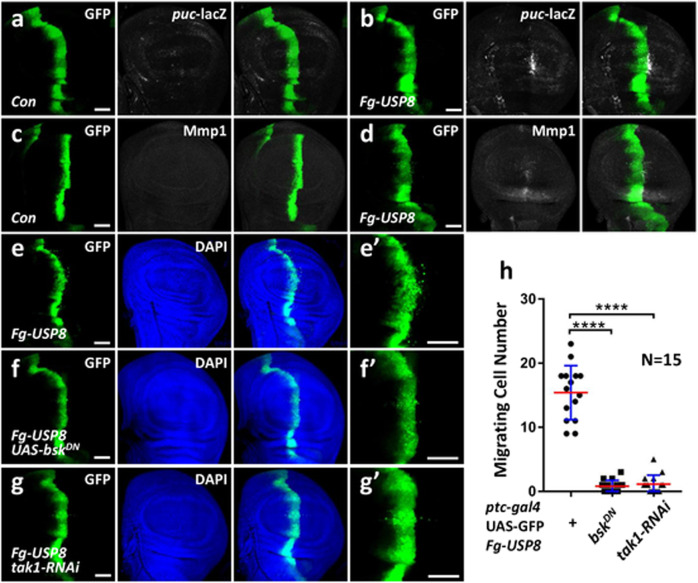
Human USP8 can replace *Drosophila* Usp8 to modulate the JNK pathway. **a** A control third-instar larvae wing disc expressing GFP under *ptc*-gal4 driver was stained to show GFP (green) and *puc*-lacZ (white). **b** Misexpression of USP8 increased *puc*-lacZ. **c** A control wing disc expressing GFP under *ptc*-gal4 driver was stained to show GFP (green) and Mmp1 (white). **d** Overexpression of USP8 increased Mmp1 protein. **e** Overexpression of USP8 driven by *ptc*-gal4 induced cell migration from A/P boundary to posterior. GFP (green) represents the expression regions of *ptc*-gal4 in the wing disc. **e'** was an enlarged figure of **e**. **f** Overexpression of *bsk*^*DN*^ could block *USP8*-induced cell migration. **g** Knockdown of *tak1* inhibited *USP8*-induced cell migration. **h** Quantification analyses of migrating cell numbers in **e**–**g** (*N* = 15). Above all, the *t*-test was used for statistical analyses, ****p < 0.0001. Scale bars: 50 μm for all disc images.

Given key components and regulatory mechanisms of the JNK pathway are evolutionarily conserved from *Drosophila* to mammals [6], we sought to test whether the effect of Usp8 on JNK signaling was also applicable to mammalian systems. Compared with the control disc (Fig. 6a), overexpression of *USP8* in the wing disc activated *puc*-lacZ expression (Fig. 6b). Furthermore, ectopic expression of *USP8* also elevated Mmp1 (Fig. 6c, d), together suggesting that USP8 is able to activate the *Drosophila* JNK pathway.

Since our above study has revealed that Usp8 promotes tumor cell migration, we next examined whether USP8 is involved in tumor cell migration. Similar to Usp8, ectopic expression of *USP8* in the wing disc triggered cell migration (Fig. 6e, h), which was attenuated by *bsk*^*DN*^ (Fig. 6f, h) or *tak1* RNAi (Fig. 6g, h). Taken together, these results suggest that USP8 can replace Usp8 to trigger cell migration in a Tak1-dependent manner.

### USP8 positively regulates metastasis of breast cancer cells

**Fig. 7:**
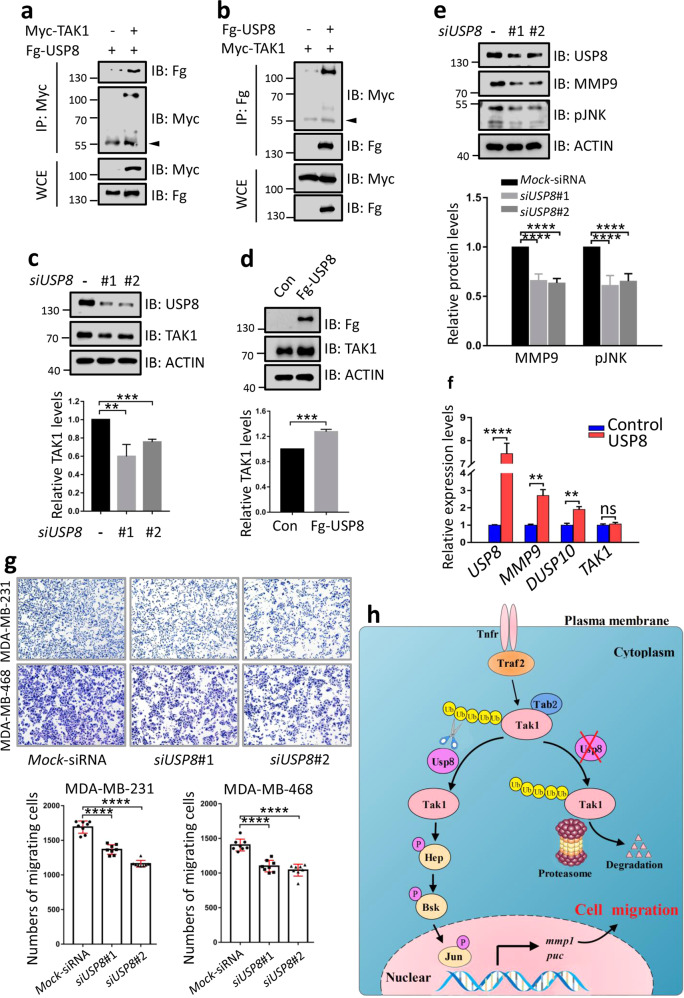
USP8 positively regulates the migration of breast cancer cells. **a** Myc-TAK1 could pull down Fg-USP8 in 293 T cells. The arrowhead indicates the IgG. **b** Fg-USP8 pulled down Myc-TAK1 in 293 T cells. The arrowhead indicates the IgG. **c** Knockdown of *USP8* decreased TAK1 protein in MDA-MB-231 cells. Quantification analyses of TAK1 protein levels are shown below (*N* = 3). **d** Overexpression of *USP8* elevated TAK1 protein in MDA-MB-231 cells. Quantification analyses of TAK1 protein level were shown below (*N* = 3). **e** Knockdown of *USP8* decreased MMP9 and pJNK protein levels in MDA-MB-231 cells. Quantification analyses of MMP9 and pJNK protein levels are shown below (*N* = 3). **f** USP8 increased mRNA levels of *MMP9* and *DUSP10* in MDA-MB-231 cells, without affecting *TAK1* mRNA level. **g** Transwell analyses showed that knockdown of *USP8* inhibited the migration of breast cancer cells. Quantification analyses of migrating cell numbers are shown below. **h** A proposed working model of Usp8 deubiquitinating Tak1 to activate the JNK signaling and cell migration. Tnfr Tumor necrosis factor receptors, Traf TNF-receptor-associated factor 2, Tak1 TGF-*β*-associated kinase 1, Tab2 TAK1-associated binding protein 2, Usp8 ubiquitin-specific protease 8, Hep Hemipterous, Bsk Basket, *puc* puckered, *mmp1* matrix metalloproteinase 1. Above all, the *t*-test was used for statistical analyses, **p < 0.01; ***p < 0.001; ****p < 0.0001 and ns no significance.

Having established that USP8 activates the JNK pathway and trigger cell migration in the *Drosophila* wing disc, we next needed to test whether USP8 modulates human tumor cell metastasis. First, co-IP assays revealed that USP8 reciprocally interacted with TAK1 in 293 T cells (Fig. 7a, b). Furthermore, endogenous USP8 was capable of pulling down endogenous TAK1 in breast cancer cells (Supplementary Fig. 5a, b). Consistently, knockdown of *USP8* decreased TAK1 protein (Fig. 7c), while overexpression of *USP8* exerted an opposite effect in MDA-MB-231 cells (Fig. 7d), together indicating that TAK1 is a possible target of USP8. Consistently, co-expression analysis conducted by the CVCDAP analysis tools (https://omics.bjcancer.org/cvcdap/home.do) showed that the protein expression level of USP8 positively correlated with TAK1 in human breast cancer samples (*N* = 105, *p* = 0.00023) (Supplementary Fig. 5c). To validate the correlation between USP8 and TAK1 expression level in breast cancer cells, we analyzed protein and mRNA levels of USP8 and TAK1 in five breast cancer cells. Results revealed that USP8 positively correlated with TAK1 in different breast cancer cells (Supplementary Fig. 5d, e). Cell-based ubiquitination experiments showed that USP8 was able to remove ubiquitin modifications from TAK1 protein in breast cancer cells (Supplementary Fig. 5g). The deubiquitination of TAK1 by USP8 was abolished by treatment with specific USP8 inhibitors (Supplementary Fig. 5g). On the other hand, overexpression of *USP8* elevated (Supplementary Fig. 5f), whereas knockdown of *USP8* decreased the expression of MMP9 (the ortholog of Mmp1 in human) and pJNK levels (Fig. 7e). In addition, quantitative real-time PCR (qRT-PCR) experiment revealed that overexpression of *USP8* promoted transcription of JNK pathway target genes, including *MMP9*, *DUSP10* (Fig. 7f). Phosphorylation and nuclear accumulation of the transcription factor c-Jun is a key step for activating the JNK pathway. Thus, nuclear phosphorylated c-Jun (P-c-Jun) serves as an indicator for judging the activity of JNK signaling. As expected, overexpression of *USP8* promoted nuclear accumulation of phosphorylated c-Jun (P-c-Jun-Ser63 and P-c-Jun-Ser73), without affecting total c-Jun levels (Supplementary Fig. 5h). These results suggest that USP8 activates the JNK signaling pathway activity through stabilizing TAK1 protein.

To investigate whether USP8 regulates tumor cell migration, transwell assays were carried out in two breast cancer cell lines (MDA-MB-231 and MDA-MB-468). In line with our speculation, overexpression of *USP8* elevated (Supplementary Fig. 6a, b), whereas knockdown of *USP8* decreased tumor cell migration (Fig. 7g). In addition, the invasion assay suggested that USP8 also promoted MDA-MB-231 cell invasion, while *USP8* siRNA exerted an opposite effect (Supplementary Fig. 6c). USP8-induced cell invasion was blunted by USP8 inhibitors (Supplementary Fig. 6c). Taken together, these data demonstrate that USP8 positively regulates metastasis of breast cancer cells, providing a potential therapeutic target for breast cancer.

## Discussion

Herein, we find that Usp8 promotes tumor cell migration through activating the JNK pathway. Genetic and biochemical analyses show that Usp8 binds Tak1 to remove polyubiquitin chains from Tak1, culminating in Tak1 stabilization and the JNK pathway activation (Fig. 7h). Furthermore, we reveal that human USP8 can replace *Drosophila* Usp8 to activate the JNK pathway and trigger cell migration. Finally, we uncovered that human USP8 positively regulates breast cancer cell migration through stabilizing TAK1. Taken together, our study reveals Usp8 is a novel regulator of the JNK pathway, provides human USP8 as a promising drug target for JNK-related breast cancer treatment.

Here, we find that Usp8 overexpression results in the loss of the anterior cross-vein (ACV) in adult wings, suggesting a physiological role of Usp8 in wing development. However, the knockdown of *usp8* in the wing fails to produce any obvious defects, possibly due to limited RNAi efficiency or functional redundancy. As a matter of fact, to overcome the shortcomings of RNAi-mediated screening, we generate transgenic flies to express *Drosophila* deubiquitinases for screening. Through this screening, we identify that Usp8 is an important modulator for JNK-mediated tumor cell migration. Given that the JNK pathway plays role in multiple physiological processes, it is also acceptable that Usp8 is involved in development. Loss of *usp8* leads to embryonic lethality [31], indicating its important role in embryogenesis. To address its physiological function, it is urgent to explore the spatiotemporal expression of *usp8* during different developmental stages.

Several substrates of Usp8 have been identified, including Smo [18, 19], Hrs [20], *α*-Synuclein [21], and CHMP1B [22]. Here, we add Tak1 as a novel substrate of Usp8. It is still unclear how Usp8 determines which substrate is stabilized, and whether distinct substrates compete to bind Usp8. Usp8 possibly recognizes substrates in context-dependent manners. The JNK pathway is a kinase cascade, which the phosphorylation is essential for the pathway activation. The phosphorylation status of Tak1 is controlled by upstream stimuli [32, 33]. The relationship between Tak1 ubiquitination and phosphorylation remains unknown.

Previous studies have demonstrated Tak1 protein undergoes distinct linkages of polyubiquitination [12, 30]. The K63-linked polyubiquitination on Tak1 is required for its autophosphorylation and subsequent activation [12], while K48-linked polyubiquitination promotes proteasome-mediated Tak1 degradation [30]. Thus, these two types of polyubiquitination bring opposite outcomes. In this study, our results show that Usp8 is able to remove both K48- and K63-polyubiquitin chains from Tak1, suggesting that Usp8 cannot distinguish distinct K48- and K63-linked polyubiquitin chains. Given that K48- and K63-linked polyubiquitination exert opposite effect, Usp8 possibly plays dual roles for regulating Tak1 activity.

Increasing studies have revealed that the elevated expression of human USP8 correlates with tumorigenesis, including proliferation and metastasis [34]. USP8 promotes the progression of breast cancer via deubiquitinating and stabilizing Connexin-43 (Cx43) and Notch1 intracellular domain (NICD) [24, 35]. Through our present results, we cautiously add that, in addition to Cx43 and NICD, TAK1 is another target of USP8 for its regulation on breast cancer progression. It will be interesting to examine whether the USP8-TAK1 axis is also applicable in other types of tumors. The previous study has revealed that the small molecule inhibitor of USP8 is able to suppress growth and metastasis of distinct tumor cells [36, 37], providing USP8 inhibitors as potential anti-tumor drugs. Although our results clearly have demonstrated that USP8 suppresses migration of breast cancer cells, we do not examine whether USP8 regulates tumorigenesis in mouse model. In the further, we will try to dissect the in vivo role of USP8 using knockin mouse model.

Taken together, our findings demonstrate that a conserved Usp8-Tak1 module promotes tumor cell migration, and provides USP8 as a potential therapeutic target for cancer treatment.

## Materials and methods

### DNA constructs

To generate Fg-Usp8, Myc-Tak1, Myc-Bsk, Myc-Hep, Myc-Traf2 and HA-Ub constructs, the corresponding coding sequences were amplified by the DNA polymerase (Vazyme, P505), and cloned them into the pcDNA3.1-Fg, pcDNA3.1-Myc or pcDNA3.1-HA backbone vectors, respectively. Truncated constructs including Usp8-N, Usp8-C, Tak1-N, Tak1-C were created by inserting the indicated coding sequences into the corresponding backbone vectors. To generate the Fg-USP8 and Myc-TAK1 constructs for mammalian cell expression, the corresponding full-length coding sequences were amplified from 293 T cell cDNA and cloned into pcDNA3.1-Fg or pcDNA3.1-Myc vectors, respectively.

### *Drosophila* stocks and genetics

The *ptc-gal4*, *Ay-gal4*, *mirr-gal4*, UAS-GFP had been described in our previous studies [38, 39]. UAS-Fg*-usp8* and *usp8*-RNAi (#5798R-1, NIG; #107623, VDRC) were kindly provided from Dr. Junzheng Zhang’s lab [20]. The *scrib* RNAi and *traf2* RNAi flies were obtained from Dr. Lei Xue’s lab [4, 40]. *puc*-lacZ (#109029, KDSC), *UAS-bsk*^*DN*^ (#9311, BDSC), *bsk* RNAi (TH04355.N, THFC), *hep* RNAi (2190R-1, NIG), *tak1* RNAi (TH04381.N, THFC) were purchased from Bloomington *Drosophila* Stock Center (BDSC), Kyoto *Drosophila* Stock Center (KDSC), Vienna *Drosophila* Stock Center (VDRC), Fly stocks of National Institute of Genetics (NIG), or Tsinghua Fly Center (THFC). The UAS-Fg-*USP8* transgenic fly was generated by injection of UAS-attB-Fg-*USP8* construct into the *Drosophila* embryos using phiC31 integration system. All the stocks were reared on standard *Drosophila* media at 25 °C. For all fly cross experiments, the indicated *gal4* virgin flies were crossed with male flies of corresponding RNAi or transgenes at 25 °C.

### Immunostaining and confocal

Immunostaining of imaginal discs were performed according to the method previously described [41]. Wing and eye imaginal discs of 3rd instar larvae were dissected in phosphate-buffered solution (PBS) and immediately fixed in freshly made 4% formaldehyde in PBS for 20 min at room temperature, then washed with PBT (PBS containing 0.1% Triton X-100) for three times. The dissected larvae were incubated with the corresponding primary antibody overnight at 4 °C, then washed three times by PBT and incubated with indicated fluorophore-conjugated secondary antibody in the dark at room temperature for 2 h. Subsequently, washed with PBT for three times, imaginal discs were separated and mounted with 40% glycerol. Images were obtained by using the Zeiss confocal microscope. Antibodies used in this study were as follows: mouse anti-Fg (1:1000; M2, Sigma), rabbit anti-*β*-Galactosidase (1:500, MBL), rat anti-Ci (1:50, DSHB), mouse anti-Mmp1 (1:5, DSHB), rabbit anti-pJNK (1:200, CST) and DAPI (1:1000, Sigma). Mouse anti-Usp8 (1:50) was generated using aa1-333 of Usp8 protein as the antigen according to the method previously described [38]. Mouse anti-Tak1 (1:50) was generated using aa500-679 of Tak1 protein as the antigen. In this study, secondary antibodies (Jackson ImmunoResearch) were used at a 1:500 dilution.

### Cell culture, transfection, immunoprecipitation, and immunoblotting

The 293 T cell line and five human breast cancer cell lines (MDA-MB-231, MDA-MB-468, MDA-MB-453, MCF-7, T47D) were purchased from the American Type Culture Collection (ATCC; Manassas, VA, USA). All the cells were cultured in a humidified incubator at 37 °C with 5% CO_2_ conditions. The 293 T cells were cultured in Dulbecco’s modified Eagle’s medium (DMEM, Gibco) supplemented with 10% FBS and 1% penicillin/streptomycin (PS). The MDA-MB-231 and MDA-MB-468 cells were cultured in DMEM/F-12 (1:1) medium (Gibco) containing 10% FBS and 1% PS. Plasmids were transfected into the 293 T and breast cancer cells using Lipofectamine 2000 (Invitrogen) according to the manufacturer’s instructions. The cells were collected 48 h after transfection and used for immunoprecipitation (IP) and immunoblotting (IB) analysis according to standard protocols. Antibodies used for IP and IB were as follows: mouse anti-Fg (1:5000 for IB, 1:500 for IP; Sigma), mouse anti-Myc (1:2000 for IB, 1:200 for IP; Santa Cruz), mouse anti-HA (1:2000 for IB, Santa Cruz), mouse anti-Actin (1:5000, Genscript), mouse anti-Usp8 (1:1000 for IB, 1:100 for IP), rabbit anti-USP8 (1:1000 for IB, 1:100 for IP; ABclonal), rabbit anti-TAK1 (1:1000 for IB, 1:100 for IP; ABclonal), rabbit anti-MMP9 (1:1000 for IB; ABclonal), rabbit anti-pJNK (1:1000 for IB; ABclonal), rabbit anti-c-Jun (1:1000 for IB; ABclonal), rabbit anti-phospho-c-Jun (Ser63) (1:1000 for IB; ABclonal), rabbit anti-phospho-c-Jun (Ser73) (1:1000 for IB; ProteinTech), rabbit anti-Lamin B (1:2000 for IB; ProteinTech), rabbit anti-*β*-tubulin (1:1000 for IB; ABclonal), goat anti-mouse HRP (1:10,000; Abmax), and goat anti-rabbit HRP (1:10,000; Abmax). USP8 inhibitor (DUB-IN-2, DUB-IN-3) was purchased from MedChem Express (Monmouth Junction, NJ, USA).

### siRNAs

To knock down *USP8* in human breast cancer cells, two different siRNAs were used respectively. The siRNAs were synthesized by GenePharma (Shanghai, China), and the sequences were as follows: *mock*-siRNA: 5ʹ-UUC UCC GAA CGU GUC ACG UdTdT-3ʹ; *USP8*-siRNA#1: 5′-GGG UCA AUU CAA AUC UAC AdTdT-3′; *USP8*-siRNA#2: 5'-ACC GAA ACU GUU AUC AGG AUG AUA UdTdT-3' [42]. siRNAs (100 nM) were transfected using Lipofectamine 2000 according to the manufacturer’s instructions. Cells were harvested after transfection with siRNAs for 72 h, and the knockdown efficiency of siRNA was detected by IB.

### GST fusion protein pull-down assay

The GST-vector and GST fusion protein plasmids were transfected into *Escherichia coli* BL-21 cells; fusion proteins were induced expression by isopropyl *β*-D-thiogalactoside (IPTG) and purified using the Beaver beads GSH (Beaverbio) as previously described [38]. GST fusion protein solutions were incubated with beads at 4 °C for 2 h. Whole-cell lysates, which contain Fg-Usp8 or Myc-Tak1 recombinant proteins, were prepared through 293 T cells. Then, GST fusion protein solution was incubated with cell lysates at 4 °C for 3 h. The bound proteins were detected by IB assay.

### Protein stability assay and ubiquitination assay

The protein stability assay was conducted as previously described [39]. In brief, 293 T cells were plated in the cell dishes (10 cm) and transfected with indicated plasmids. After transfection for 24 h, cells of equal density and volume were transferred into 12-well cell culture plates. Cells were treated with cycloheximide (CHX, 20 μg/ml) to inhibit the protein synthesis for indicated times before harvesting. IB was performed to detect the levels of indicated proteins. Cells were co-transfected with HA-Ub and indicated plasmid, and the proteasome inhibitor MG132 (50 μM for 4 h) was added into the medium prior to harvest. Subsequently, immunoprecipitation was conducted to obtain ubiquitinated Myc-Tak1, and the levels of indicated proteins were detected by IB assay.

### RNA isolation and quantitative real-time PCR

Total RNA was isolated from breast cancer cells by TRIzol reagent (Invitrogen) according to the standard protocols. The cDNA template was synthesized using 1 µg of total RNA by HiScript^®^ Q RT SuperMix with gDNA wiper (Vazyme) following the manufacturer’s instructions. The quantitative real-time PCR (qRT-PCR) experiment was conducted with ChamQ SYBR^®^ Color qPCR Master Mix (Vazyme) by the LightCycler^®^ 96 System (Roche Molecular Biochemicals, Lewes, United Kingdom). The primers used for qRT-PCR were as follows: *ACTIN*, 5'-GAT CAT TGC TCC TCC TGA GC-3' (forward) and 5'-ACT CCT GCT TGC TGA TCC AC-3' (reverse), *USP8*, 5'-TTC CAT TCA ATA CTT GGA CCT GG-3' (forward) and 5'-CCA AAG AGC CTT TAG CCA ATG T-3' (reverse), *TAK1*, 5'-CCG GTG AGA TGA TCG AAG CC-3' (forward) and 5'-GCC GAA GCT CTA CAA TAA ACG C-3' (reverse), *DUSP10*, 5'-ATC GGC TAC GTC ATC AAC GTC-3' (forward) and 5'-TCA TCC GAG TGT GCT TCA TCA-3' (reverse), *MMP9*, 5'-AGA CCT GGG CAG ATT CCA AAC-3' (forward) and 5'-CGG CAA GTC TTC CGA GTA GT-3' (reverse). Relative quantification was calculated by the comparative 2^−ΔΔCt^ method [43], and the values are shown as the mean ± SD (standard deviation).

### Transwell Assays

The MDA-MB-231 or MDA-MB-468 cells (1 × 10^5^ cells) were planted into the upper chamber with serum-free medium after transfection for 48 h. Meanwhile, a medium with 10% FBS was added in the lower chamber. Then, the cells were cultured at 37 °C for 12 h (MDA-MB-231 cells) or 48 h (MDA-MB-468 cells). Migrated cells were fixed with 20% methanol for 20 min and stained with crystal violet (0.1%). The invasion assay was performed using the tissue culture plate inserts (TCS003024) (Guangzhou Jet Bio-Filtration Co., Ltd., Guangzhou, China) according to the manufacturer’s instructions. The image was acquired under a light microscope and the numbers of migrating cells were counted by the Image J software.

### Statistical analysis

Quantification of the band intensity of immunoblotting was performed using the Image J software. Statistical analysis was conducted by the software of GraphPad Prism 7.0 (GraphPad Software Inc., San Diego, CA). All the data shown in the figures were representative of at least three independent replicates and shown as the means ± SD. Unpaired student’s *t*-test was employed for group comparisons. *P* < 0.05 was considered to be statistically significant (*P < 0.05, **P < 0.01, ***P < 0.001, ****P < 0.0001, ns: not significant).

## Supplementary information

Supplementary materials

reproducibility-checklist

## Data Availability

All relevant data mentioned in this manuscript are available from the corresponding author upon reasonable request.
