# Supplementary material for: Usp8 promotes tumor cell migration through activating the JNK pathway
Source: Cell Death Dis. 2022 Mar 31;13(3):286. doi: 10.1038/s41419-022-04749-1 (PMC8971431; doi:10.1038/s41419-022-04749-1)

Supplementary materials

Usp8 promotes tumor cell migration through activating the JNK pathway

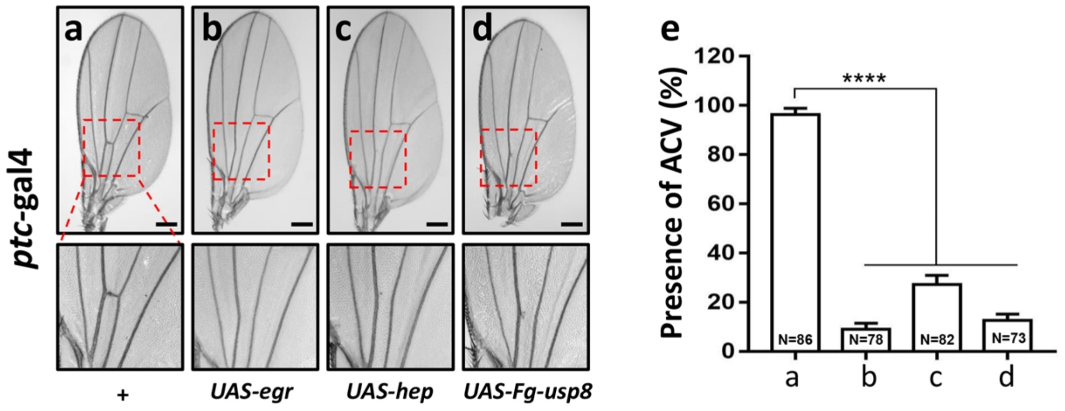

Supplementary Fig. 1 Ectopic expression of Usp8 resulted in loss of the ACV.

a The adult wing of *ptc*-gal4 was used as a control. **b, c** Overexpression of *egr* or *hep*, two well-known positive regulators of the JNK pathway, led to the loss of ACV. **d** Overexpression of *usp8* resulted in the loss of ACV. **e** Statistical analyses of the presence of ACV in **a-d** were presented as mean ± SD. Numbers of wings for statistical analyses were shown in columns. The *t*-test was used for statistical analyses, $****p < 0.0001$.

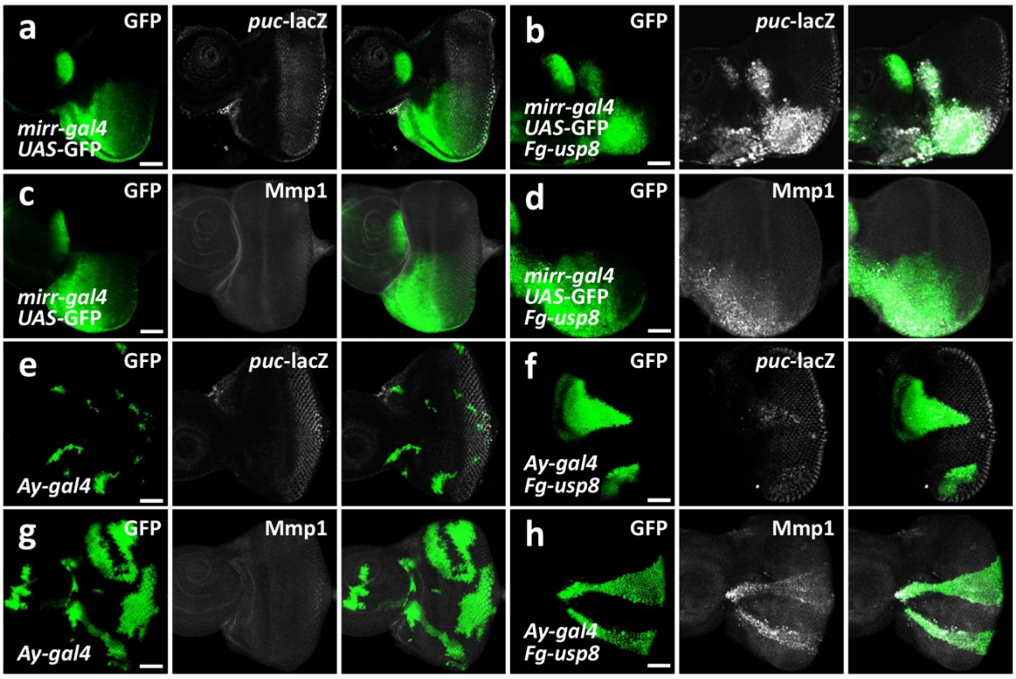

Supplementary Fig. 2 Ectopic expression of *usp8* activates the JNK in eye discs.

a A control third-instar larvae eye disc expressing GFP under *mirr*-gal4 driver was stained to show GFP (green) and *puc*-lacZ (white). **b** Overexpression of *usp8* by *mirr*-gal4 increased *puc*-lacZ. **c** A control eye disc expressing GFP under *mirr*-gal4 driver was stained to show GFP (green) and Mmp1 (white). **d** Overexpression of *usp8* increased Mmp1 protein. **e** A control eye disc expressing GFP via FLP-out technique was stained with GFP (green) and *puc*-lacZ (white). **f** Ectopic expression of *usp8* in the eye disc by FLP-out technique elevated *puc*-lacZ. **g** A control eye disc expressing GFP via FLP-out technique was stained with GFP (green) and Mmp1 (white). **h** Overexpression of *usp8* in the eye disc by FLP-out technique elevated Mmp1 protein. Scale bars: 50μm for all disc images.

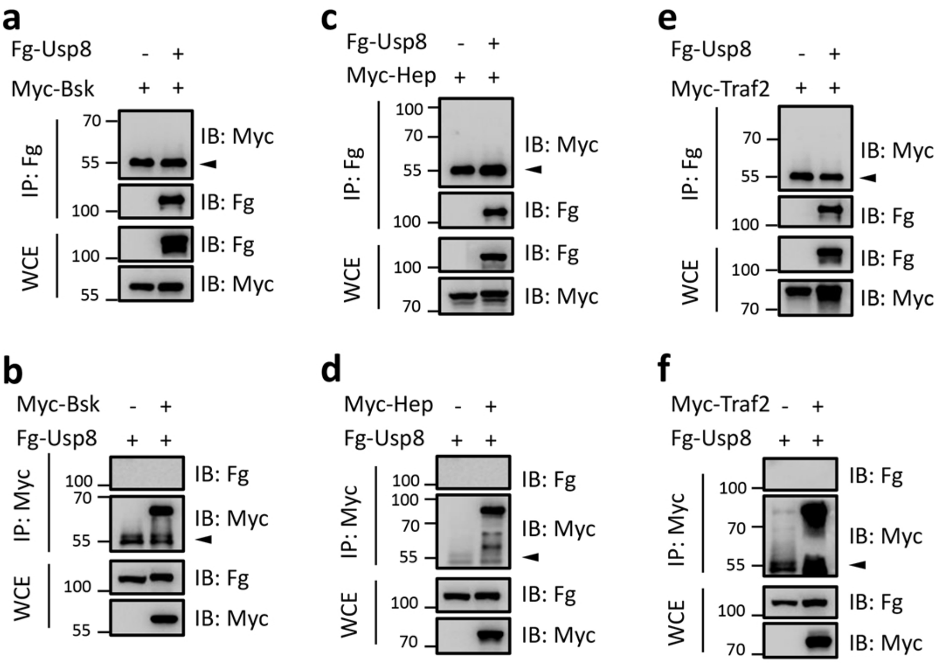

Supplementary Fig. 3 Usp8 cannot interact with other key components of the JNK pathway.

a, b Fg-Usp8 did not interact with Myc-Bsk. **c**, **d** Fg-Usp8 was unable to bind Myc-Hep. **e, f** Fg-Usp8 failed to pull down Myc-Traf2. Above all, arrowheads mark the IgG bands.

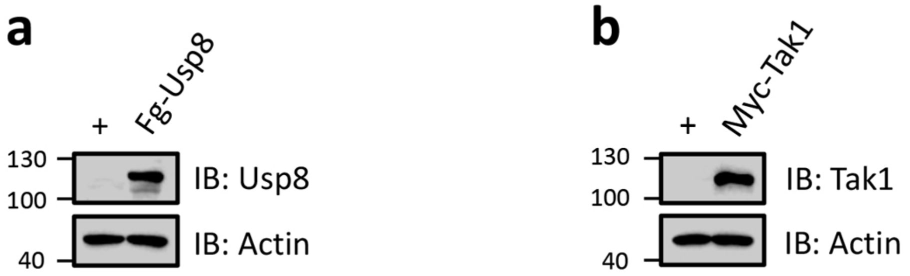

Supplementary Fig. 4 Usp8 and Tak1 antibodies work well.

a Mouse anti-Usp8 antibody could recognize exogenous Fg-Usp8 protein. Fg-Usp8 construct was introduced into 293T cells. **b** Mouse anti-Tak1 antibody could recognize exogenous Myc-Tak1 protein. Myc-Tak1 construct was introduced into 293T cells.

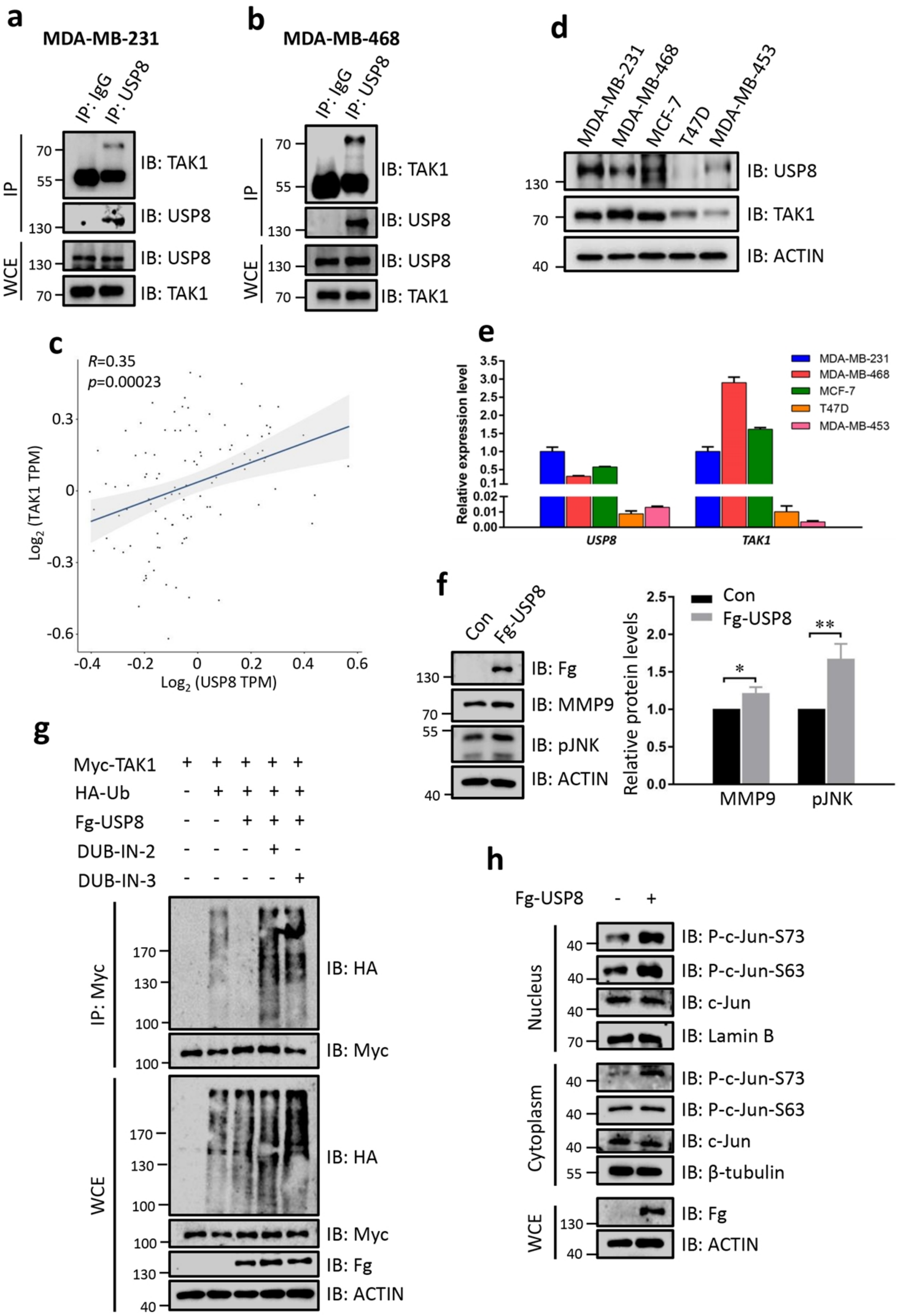

Supplementary Fig. 5 USP8 interacts with TAK1, and activates the JNK pathway in breast cancer cells.

a, b Endogenous USP8 interacted with endogenous TAK1 in breast

cancer cells (**a**, MDA-MB-231; **b**, MDA-MB-468). **c** The protein co-expression analysis of USP8 and TAK1 was conducted using the CVCDAP analysis tools. Of note, USP8 positively correlated with TAK1 in human breast samples. **d, e** Immunoblotting (IB) (**d**) and qRT-PCR (**e**) analyses were used to detect protein and mRNA levels of USP8 and TAK1 in five breast cancer cell lines. **f** Overexpression of *USP8* increased MMP9 and pJNK protein levels in MDA-MB-231 cells. Quantification analyses were shown on the right (N=3). **g** Immunoblots of immunoprecipitates and lysates from MDA-MB-231 cells expressing indicated constructs and treated by MG132 (50 μM) and USP8 inhibitor (DUB-IN-2 and DUB-IN-3, 3 μM) for 4h before cell harvesting. Notably, USP8 attenuates TAK1 ubiquitination in breast cancer cells. **h** Immunoblots of nuclear protein and cytoplasmic protein of MDA-MB-231 cells transfected with indicated constructs. Lamin B acts as a loading control for nuclear protein, whereas β-tubulin is a loading control for cytoplasmic protein. Notably, USP8 promotes phosphorylated c-Jun (P-c-Jun-Ser63 and P-c-Jun-Ser73) accumulation in the nucleus. Above all, the *t*-test was used for statistical analyses, $^{*}p < 0.05$; $^{**}p < 0.01$.

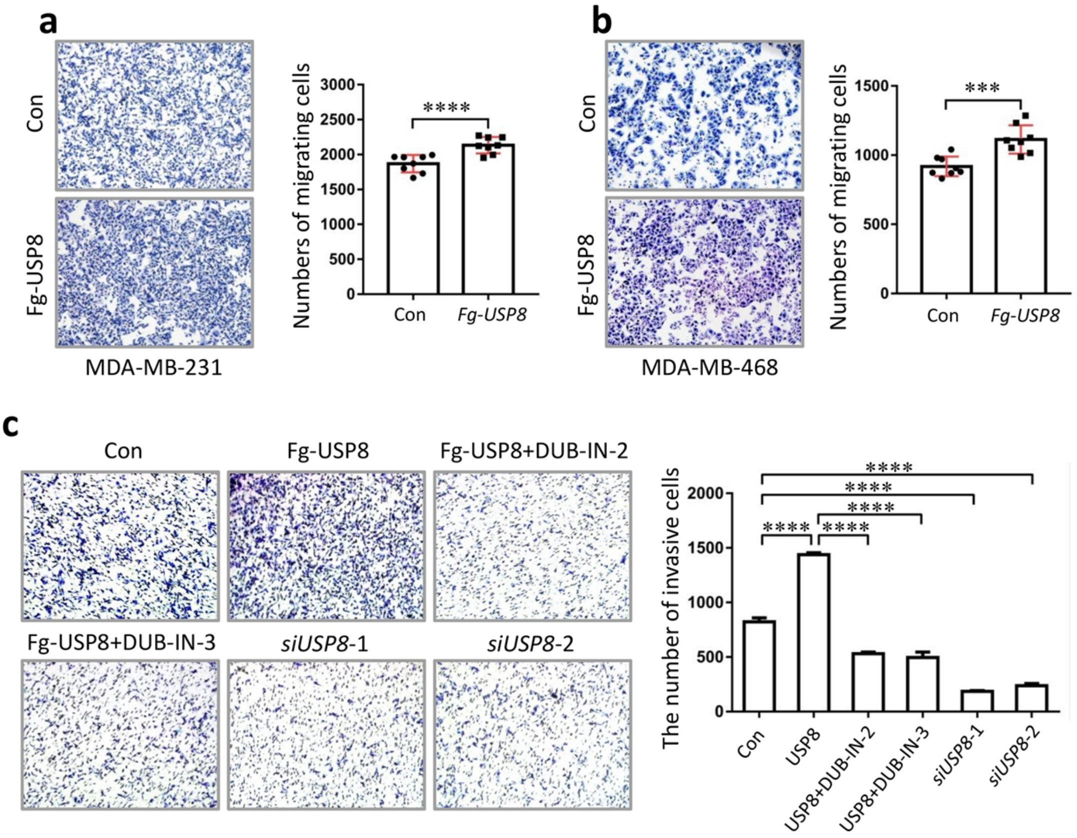

Supplementary Fig. 6 Overexpression of *USP8* promotes migration and invasion of breast cancer cells.

a, b Transwell analyses showed that overexpression of *USP8* enhanced the migration of breast cancer cells (**a**, MDA-MB-231; **b**, MDA-MB-468). Quantification analyses of migrating cell numbers were shown on the right. **c** The invasion abilities of MDA-MB-231 cells with indicated treatments were evaluated by using a Tissue Culture Plate Inserts Invasion Chamber. The USP8 inhibitor DUB-IN-2 and DUB-IN-3 (3 μM for 4h) was added into the medium prior to invasion. Above all, the *t*-test was used for statistical analyses, ***$p < 0.001$; ****$p < 0.0001$.

Original western blots

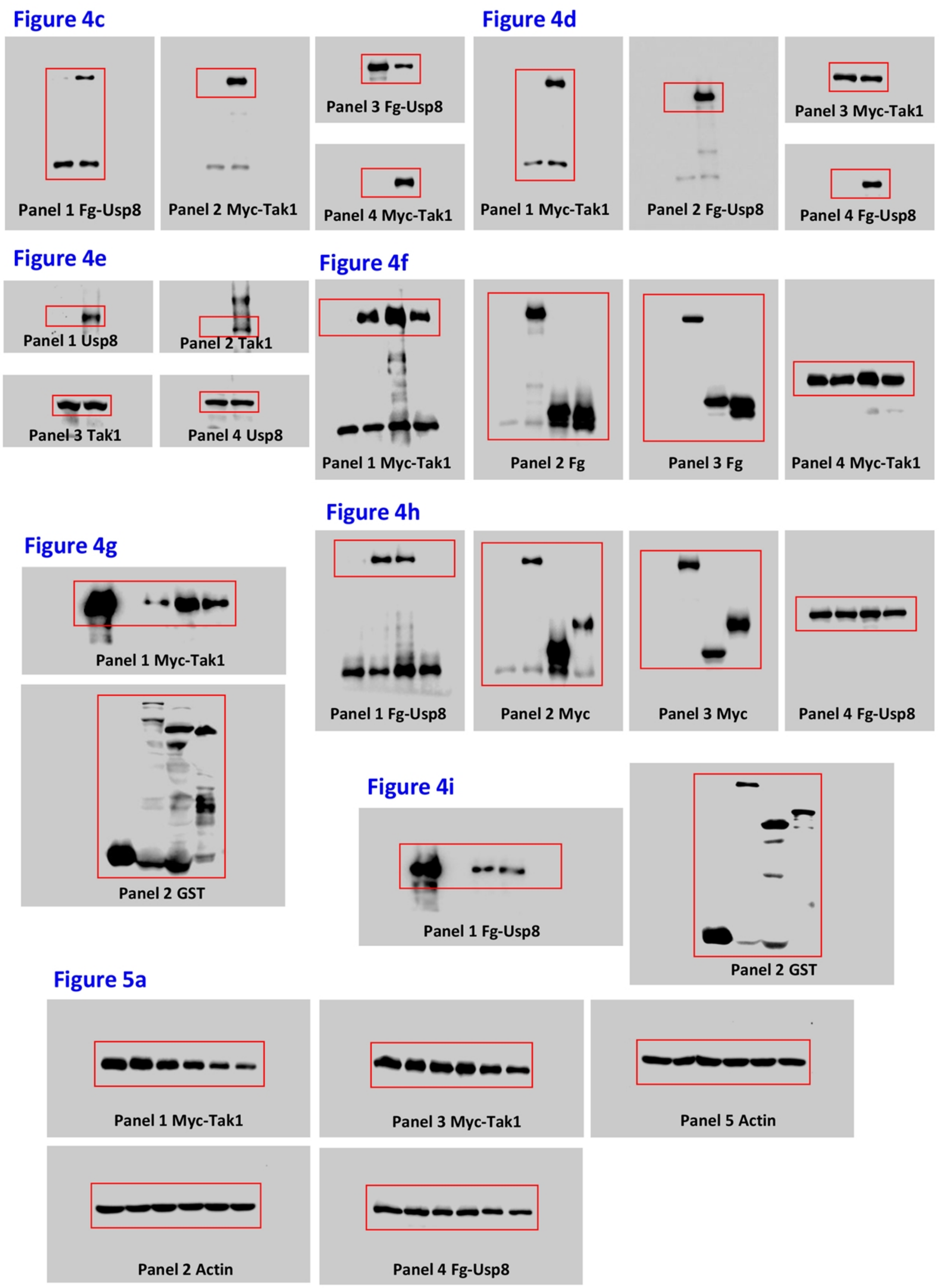

Figure 5c

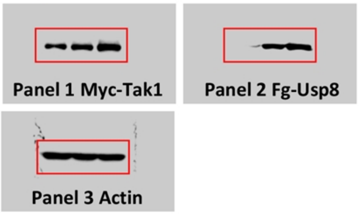

Figure 5d

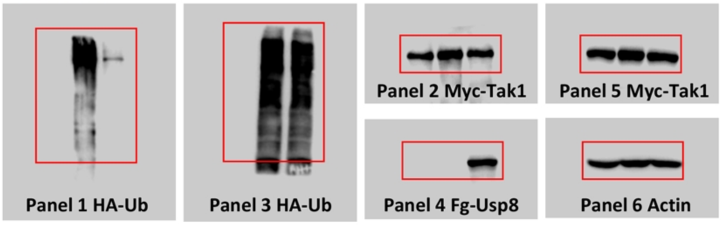

Figure 5e

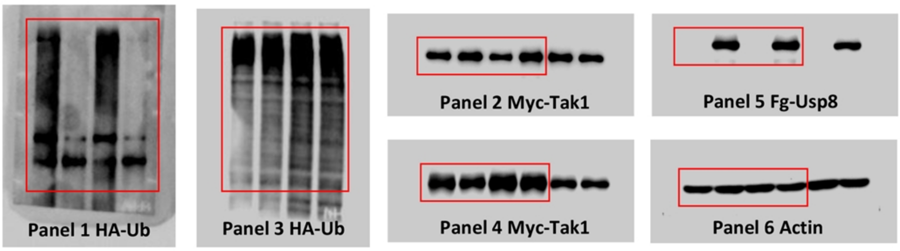

Figure 7a

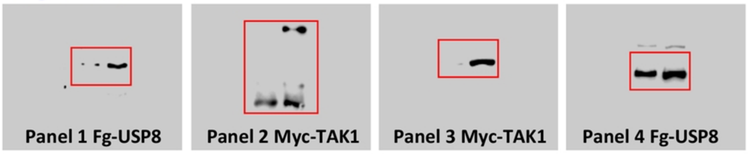

Figure 7b

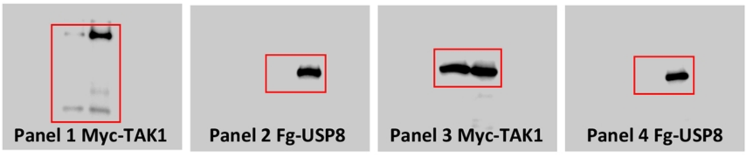

Figure 7c

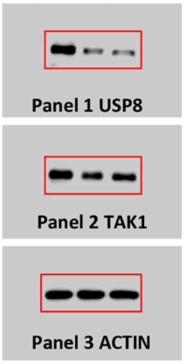

Figure 7d

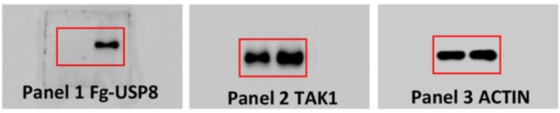

Figure 7e

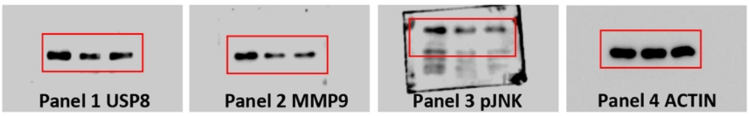

Figure S3a

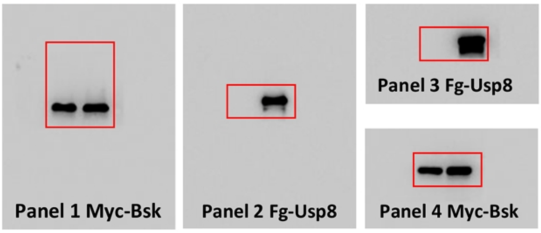

Figure S3b

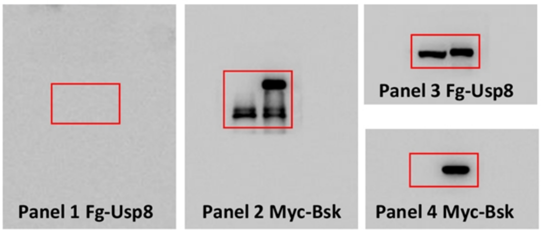

Figure S3c

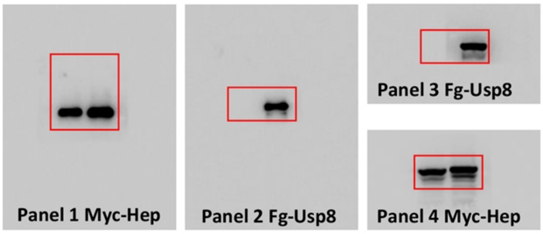

Figure S3d

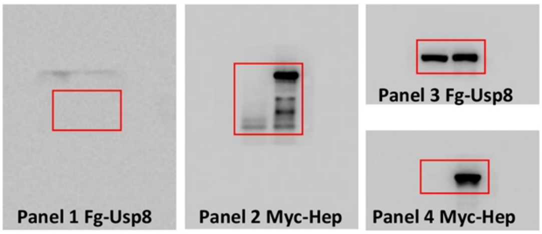

Figure S3e

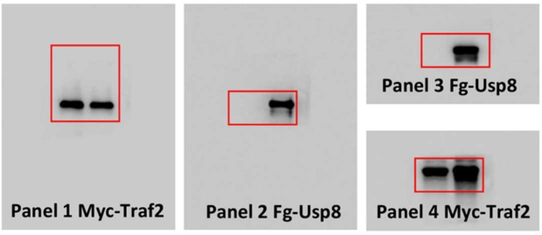

Figure S3f

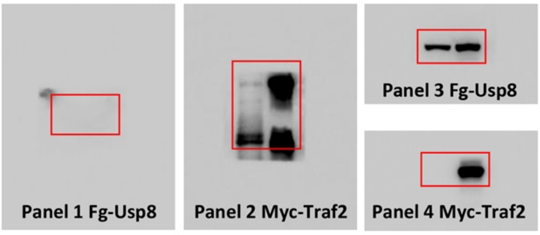

Figure S4a

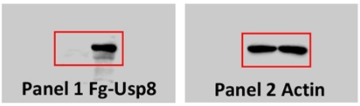

Figure S4b

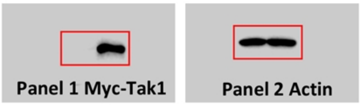

Figure S5a

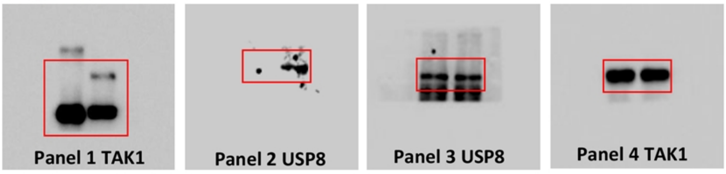

Figure S5b

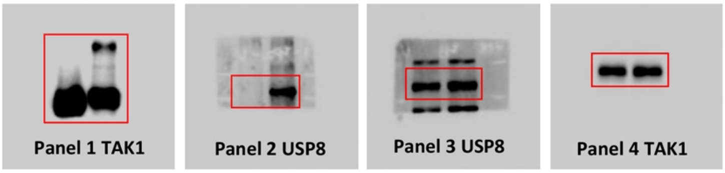

Figure S5c

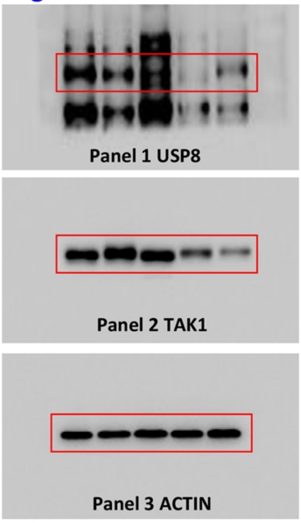

Figure S5f

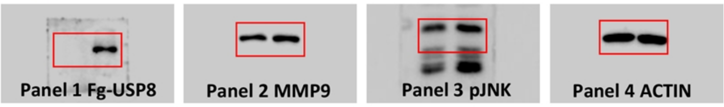

Figure S5g

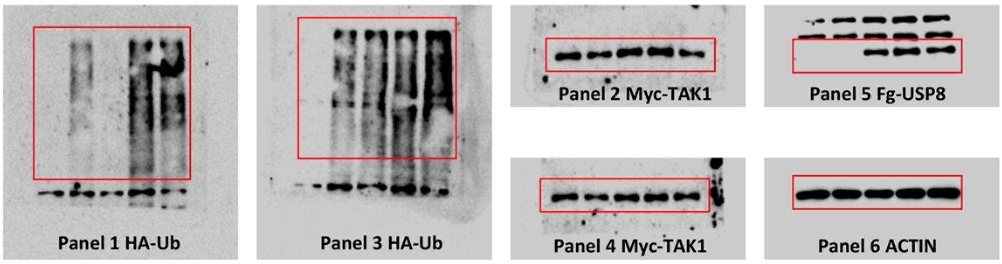

Figure S5h

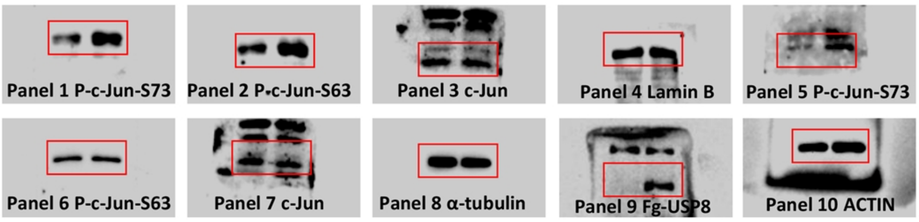

Supplement: Supplementary file 1 — Supplementary materials [file 41419_2022_4749_MOESM1_ESM.pdf]
